# Use of an EZ-Tn*5*-Based Random Mutagenesis System to Identify a Novel Toxin Regulatory Locus in *Clostridium perfringens* Strain 13

**DOI:** 10.1371/journal.pone.0006232

**Published:** 2009-07-14

**Authors:** Jorge E. Vidal, Jianming Chen, Jihong Li, Bruce A. McClane

**Affiliations:** 1 Department of Microbiology and Molecular Genetics, University of Pittsburgh School of Medicine, Pittsburgh, Pennsylvania, United States of America; 2 Center for Vaccine Research, University of Pittsburgh School of Medicine, Pittsburgh, Pennsylvania, United States of America; 3 Australian Research Council Centre of Excellence in Structural and Functional Microbial Genetics, Department of Microbiology, Monash University, Melbourne, Victoria, Australia; Columbia University, United States of America

## Abstract

**Background:**

Although useful for probing bacterial pathogenesis and physiology, current random mutagenesis systems suffer limitations for studying the toxin-producing bacterium *Clostridium perfringens*.

**Methodology/Principal Findings:**

An EZ-Tn*5*-based random mutagenesis approach was developed for use in *C. perfringens*. This mutagenesis system identified a new regulatory locus controlling toxin production by strain 13, a *C. perfringens* type A strain. The novel locus, encoding proteins with homology to the AgrB and AgrD components of the Agr quorum sensing system of *Staphylococcus aureus* and two hypothetical proteins, was found to regulate early production of both alpha toxin and perfringolysin O (PFO) by strain 13. PFO production by the strain 13 Δ*agr*B mutant could be restored by genetic complementation or by physical complementation, i.e. by co-culture of the strain 13 Δ*agr*B mutant with a *pfo*A mutant of either strain 13 or *C. perfringens* type C CN3685. A similar AgrB- and AgrD-encoding locus is identifiable in all sequenced *C. perfringens* strains, including type B, C, D, and E isolates, suggesting this regulatory locus contributes to toxin regulation by most *C. perfringens* strains. In strain 13, the *agrB* and *agrD* genes were found to be co-transcribed in an operon with two upstream genes encoding hypothetical proteins.

**Conclusions/Significance:**

The new Tn*5*-based random mutagenesis system developed in this study is more efficient and random than previously reported *C. perfringens* random mutagenesis approaches. It allowed identification of a novel *C. perfringens* toxin regulatory locus with homology to the Agr system of *S. aureus* and which functions as expected of an Agr-like quorum sensing system. Since previous studies have shown that alpha toxin and perfringolysin O are responsible for strain 13-induced clostridial myonecrosis in the mouse model, the new *agr* regulatory locus may have importance for strain 13 virulence.

## Introduction

*Clostridium perfringens* is a major pathogen of humans and other animals, causing a spectrum of serious enteric and histotoxic infections ranging from clostridial myonecrosis to *Clostridium perfringens* type A food poisoning [1]. The virulence of this Gram-positive, spore-forming anaerobe is largely attributable to its prodigious toxin production, with the literature reporting at least 17 different *C. perfringens* toxins [1]. However, toxin production varies from strain-to-strain, allowing individual *C. perfringens* isolates to be classified into types A–E, based upon their production of four typing toxins (alpha, beta, iota and epsilon toxins).

Besides being an important pathogen, *C. perfringens* is also ubiquitously distributed in the environment [1]. This bacterium is commonly found amongst the normal intestinal flora of most animal species, including humans [1], [2]. *C. perfringens* is also a common inhabitant of soils, both in its spore and vegetative forms [3]. Due to its presence in feces and ability to form resistant spores, *C. perfringens* has been used as an indicator organism for fecal water pollution [4].

*C. perfringens* is the most genetically tractable of all pathogenic clostridial species. Using allelic exchange-based techniques, it has been possible for >15 years to construct directed null mutants in transformable strains of this bacterium [5]. More recently, adaptation of group II introns (Targetrons) has greatly improved the efficacy of directed *C. perfringens* mutant construction [5]–[7]. For example, Targetron technology facilitated rapid construction of several *C. perfringens* single and double toxin null mutants [6], [8], [9], or mutants unable to express an acid soluble protein important for spore resistance properties [10], providing new understanding of *C. perfringens* virulence, pathogenesis and physiology.

Despite these recent improvements in directed mutant construction, there are still technical limits for performing genetics in *C. perfringens*. For example, random mutagenesis is a powerful tool for providing insights into bacterial gene function, but this technique remains suboptimal for all pathogenic clostridial species, including *C. perfringens*. While, Tn*916* has been successfully used for many years as a mutagenic tool for *C. perfringens* [11]–[13], this transposon suffers from several significant limitations. One problem is Tn*916'*s tendency towards multiple insertions, with several studies detecting multiple Tn*916* insertions in 65–75% of all isolated *C. perfringens* mutants [12], [13]. In addition, Tn*916* remains active after integration, resulting in unstable mutants [14]. A final problem is that Tn*916* insertion can be followed by a deletion event that removes DNA regions, rather than inactivating a specific gene [12].

Because of those Tn*916* limitations, there is interest in identifying alternative transposon tools for *C. perfringens* random mutagenesis. To that purpose, another group recently reported development of a phage Mu-based, random transposition mutagenesis system for *C. perfringens* [15], which proved relatively efficient and produced mutants with only a single transposon insertion. Although not directly addressed in that study, *C. perfringens* phage Mu-system mutants should also be stable based upon results with other bacteria [16]. However, the new phage Mu system still exhibited limitations when used in *C. perfringens*, including, i) nearly half of all the obtained mutants carried their transposon insertion in a rRNA gene [15] and ii) the number of *C. perfringens* mutants obtained per µg of Erm-Mu transposase DNA was relatively low, e.g., 239 transformant colonies/µg DNA for strain JIR325 (a strain 13 derivative) [15].

EZ-Tn*5* random mutagenesis (Epicentre®) is an alternative approach to phage Mu-based systems for random mutagenesis. The EZ-Tn*5* system has allowed random mutagenesis in many bacterial species (www.epibio.com) and retains the simplicity of phage Mu-based mutagenesis. However the EZ-Tn*5* system possesses two important advantages, i) a high transposition frequency (e.g., reportedly ∼100-fold higher than phage Mu-based systems) and ii) the highest degree of “randomness” for insertions among commercially-available transposition systems, including phage Mu (www.epibio.com).

**Figure 1:**
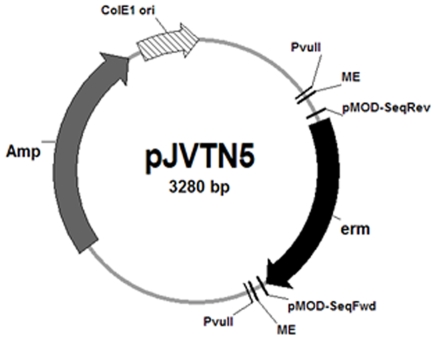
Modification of the EZ-Tn*5*-encoding vector for random mutagenesis in *C. perfringens*. To allow selection of *C. perfringens* transformants after electroporation with the EZ-Tn*5* transposon, a *C. perfringens* erythromycin resistance determinant (*erm*) was cloned into the multiple cloning site in the Epicentre EZ-TN*5*-encoding pMOD-2 vector creating pJVTN5. This plasmid also contains PvuII-recognized sequences flanking the mosaic end (ME) sites, which are specifically recognized by the EZ-Tn*5* transposase.

Transformants carrying a EZ-Tn*5* insertion are easily identifiable by antibiotic selection. However, commercial EZ-Tn*5* transposons are sold with antibiotic resistance determinants that either do not work in *C. perfringens* or would impart novel resistance characteristics to this organism. Therefore, to explore the potential usefulness of the EZ-Tn*5* system for improving random mutagenesis in *C. perfringens*, we modified a commercial EZ-Tn*5* pMOD vector by inserting a *C. perfringens* erythromycin resistance determinant (Fig. 1). This modified EZ-Tn*5* transposon proved highly useful for random mutagenesis of *C. perfringens*, identifying a novel locus regulating the production of both α toxin and perfringolysin O (PFO) in strain 13.

## Results

### Development of an EZ-Tn*5*-based random mutagenesis system for *C. perfringens*

A transposome mixture (containing the *erm-*carrying transposon DNA+EZ−Tn*5* transposase) was electroporated into strain 13. A total of ∼280 erythromycin (Erm)-resistant transformants were obtained in each of three independent transformation experiments with strain 13. PCR reactions using primers erm-Fwd-EcoRI and erm-Rev-HindIII confirmed that the *erm* gene, which is present in the modified Tn*5* transposon but is not naturally encoded by *C. perfringens* strain 13, was carried by all strain 13 transformants growing on BHI plates containing Erm (data not shown). Thus, for strain 13, the efficiency of the *erm*-carrying EZ-Tn*5* transposon insertion was ∼11,200 transformants/µg transposon DNA.

### Random insertion of the *erm*-carrying transposon in *C. perfringens*

**Figure 2:**
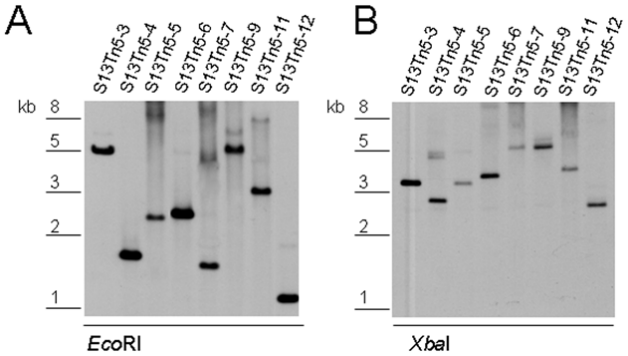
Southern blot analyses of *C. perfringens* random mutants obtained after electroporation with EZ-Tn*5* transposomes. After selection on BHI plates containing Erm (40 µg/ml), DNA was extracted from strain 13 transformants. Following digestion with EcoRI (A) or XbaI (B), the digested DNA was electrophoresed and blotted to a nylon membrane. DNA on the membranes was then hybridized with a Dig-labeled *erm* probe, as found in the *C. perfringens*-modified EZ-Tn5, and blots were developed as described in the Materials and Methods. Size of DNA fragments, in kilobases (kb), is shown at left.

Southern hybridization with an *erm* probe was used to assess the “randomness” of transposon insertion in the strain 13 mutants. Only a single copy of the *erm* gene was detected in 8 different *C. perfringens* strain 13 transposon mutants (Fig. 2). These Southern blot analyses also suggested that the *erm* gene had apparently inserted into different DNA regions in these mutants (Fig. 2). To confirm that EZ-Tn*5* insertions are random, upstream and downstream regions flanking the transposon were sequenced for 11 arbitrarily selected *C. perfringens* strain 13 mutants. Table 1 results confirmed that the transposon insertions were random and had occurred within ORF sequences, including ORFs encoding a putative virulence factor (9%), hypothetical proteins (27%), genes encoding proteins of metabolic pathways or protein biosynthesis (45%) and rRNA genes (18%).

**Table 1:** Target gene of the EZ-Tn5 based transposon in *C. perfringens* strain 13.

Mutant	Gene name or locus tag, description
S13Tn5-01 (CPJV501)	CPE1561 (*agr*B), quorum sensing regulatory protein
S13Tn5-03	rrnB-16S (CPEr004), 16S ribosomal RNA,
S13Tn5-04	CPE2407, elongation factor Tu
S13Tn5-05	CPE1142, hypothetical protein
S13Tn5-06	16S RNAr
S13Tn5-07	CPE1892, 50S ribosomal protein L20
S13Tn5-09	CPE1736, ribulose phosphate 3-epimerase
S13Tn5-10	CPE0027, hypothetical protein
S13Tn5-11	CPE2524, methionyl-tRNA synthetase
S13Tn5-12	CPE0643, hypothetical protein
S13Tn5-13	CPE2348, phosphate butyryltransferase

### Disruption of the *C. perfringens agr*B gene using EZ-Tn*5* transposon mutagenesis

To demonstrate the usefulness of the new EZ-Tn5 system, our strain 13 mutant library was screened by growth on blood agar plates or egg yolk agar plates for reduced or lost PFO-induced β-hemolysis or CPA phospolipase activity, respectively. One EZ-Tn*5*-carrying mutant, named CPJV501, exhibited a complete loss of PFO-induced β-hemolysis halo when growing on blood agar plates and a reduced phospholipase C (alpha toxin)-induced halo when growing on egg yolk agar plates (data not shown). PCR analyses, using primers shown in Table 2, indicated (data not shown) that the transposon present in this mutant had not disrupted either its *pfo*A gene (including its promoter and *vir*R boxes), the *plc* gene, or the *vir*S/*vir*R operon that had previously been shown to regulate *pfo*A and *plc* transcription [11], [17], [18].

**Table 2:** Primers used in this study.

Primer	Sequence	Reference
erm-Fwd-EcoRI	AAGGGAATTCCTAAAAATTTGTAATTAAGAAGGAGT	This study
erm-Rev-HindIII	AAGGAAGCTTCCAAATTTACAAAAGCGACTCATA	
pMOD-SeqFwd	GCCAACGACTACGCACTAGCCAAC	Epicentre
pMOD-SeqRev	GAGCCAATATGCGAGAACACCCGAGAA	
agrBFwd	GATTGAGAATATATCGAAGTTAAT	This study
agrBRev	TATGTAGGTTAGAGTCATACATTGC	
agrBF	TTACGAATTCGATGTTAGCCATGTATGCTTTCG	This study
agrBR	TAGAGGATCCTCATTTAACTCATCCCCTCAAG	
agrF1	TTACGAATTCTTAGCTCTTTATATTGGATATACAG	This study
agrR1	TAGAGGATCCCCGGTTTAAAACCGACCTTTAG	
pfoAF1	ATCCAACCTATGGAAAAGTTTCTGG	[22]
pfoAR1	CCTCCTAAAACTACTGCTGTGAAGG	
cpaF	GCTAATGTTACTGCCGTTGA	[45]
cpaR	CCTCTGATACATCGTGTAAG	
polCJVL	AATATATGATACTGAAGAGAGAGTAA	This study
polCJVR	TCTAAATTATCTAAATCTATGTCTACT	
agr101L	TAAATTTGCTCCAGTAGATACTAA	This study
agrDR	TATTCATCTCTTAAAGATTTTGGT	
agr102L	TTCAAGTTTGATATTGGTATTAGT	This study
agr101R	CAAAGCTTCTAAAGCTATATTAAA	
agr103L	ATGATAGGAACAAGTACAGTAAAA	This study
agr102R	AACTTGAAATTAAATATTCCTTCT	
agr104L	AAATTTAAAACTTGTTATTGGAGT	This study
agr103R	GGCTTTAAACTATATCCTTTTATT	
pfoA81L	CCCAGTTATTCACGATTAAAG	This study
pfoA82R	AGTAATACTAGATCCAGGGTATAAA	

**Figure 3:**
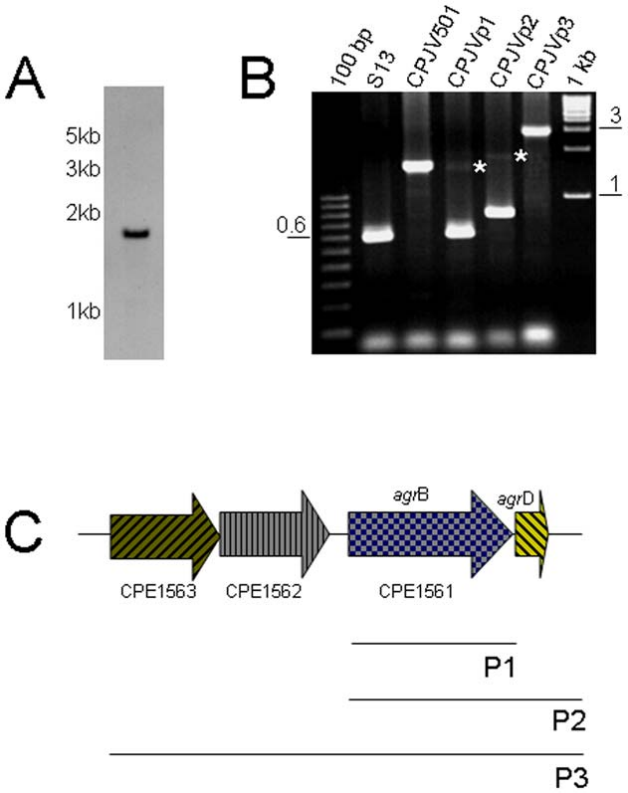
Generation of a *C. perfringens agrB* mutant and complementing strains. A) Southern blot analyses, as described in Fig. 2, using EcoRI-digested DNA from CPJV501 and a Dig-labeled probe that detected a single copy of the *erm* gene. Size of DNA fragments, in kilobases (kb) is shown at left. B) PCR was performed with DNA extracted from the indicated strain and the following pair of primers, agrBFwd and agrBRev in reactions containing DNA from strain 13 (S13), CPJV501 and CPJVp1; agrBFwd and argDR for CPJVp2 and agrF1 and agrD100R for CPJVp3. DNA ladders (100 bp or 1 kb) were included in the first and last lane of the gel. Asterisks show the expected PCR product when the primers amplified the Tn5-disprupted *agr*B gene. C) Genes cloned in the *E. coli-C. perfringens* shuttle plasmid pJIR750 to complement the *agr*B transposon mutant. As shown, P1 encodes the *agr*B gene alone, P2 the *agr*B and *agr*D genes and P3 encodes two-genes (CPE1562 and CPE1563) upstream the *agr*B gene (CPE1561) and *agr*B and *agr*D.

Therefore, the DNA flanking the *erm*-modified EZ-Tn*5* transposon in CPJV501 was sequenced, which revealed that the transposon had inserted into strain 13 ORF CPE1561 (Table 1). Southern blot analyses showed that this mutant carries only one copy of the transposon integrated in its genome (Fig. 3A). PCR analysis using primers that amplify the wt CPE1561 gene (642 bp) in strain 13 then further demonstrated the presence of the transposon-disrupted CPE1561 gene (1600 bp) in CPJV501 (Fig. 3B).

A homolog of the putative protein encoded by the CPE1561 gene has been annotated as AgrB for *C. perfringens* strain ATCC 13124 [19], which is found in other Gram-positive bacteria, including some other clostridial species. In *S. aureus*, AgrB and the secreted peptide AgrD are part of a well-characterized quorum sensing (QS) system that is involved in regulating the expression of virulence genes, including several toxins [20]. Bioinformatics analyses using the Pathema website further revealed that a sequence with 100% homology to the *agrB* ORF in *C. perfringens* strain 13 and ATCC 13124 is also present in all other sequenced *C. perfringens* isolates, including those classifying as types A–E (data not shown). Moreover, the region between ORFs CPE1561 and CPE1560 in strain 13 contains a small (135 bp) ORF (Fig. 3C) sharing ∼99% homology with a putative *agr*D gene from *C. perfringens* type A strain SM101 and ATCC13124 [21].

### The *C. perfringens agr* locus regulates PFO production

**Figure 4:**
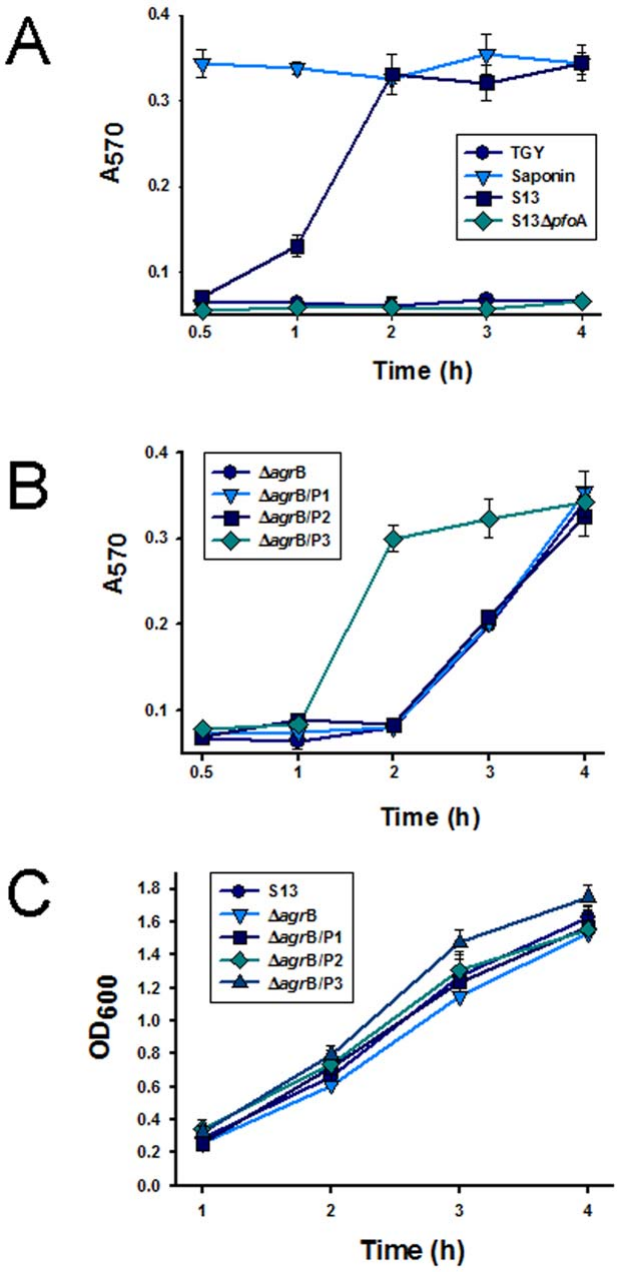
The *C. perfringens agr*B locus regulates PFO production. A and B) Hemoglobin (Hb) release assay. Culture supernatants obtained, at the indicated time point, from strain 13 (S13), S13 *pfo*A-null mutant (S13Δ*pfo*A), CPJV501 (Δ*agr*B), CPJVp1 (Δ*agr*B/P1), CPJVp2 (Δ*agr*B/P2) or CPJVp3 (Δ*agr*B/P3), were incubated (1∶1) with a 1% suspension of horse red blood cells for 30 min at 37°C. Non-inoculated TGY or 0.1% saponin (Saponin) was included as negative or positive control, respectively. PFO-induced Hb release was detected by obtaining the absorbance at 570 (A_570_). C) For each time point, the OD_600_ of the cultures is shown. For all panels, error bars represent the standard error of the mean calculated using data from three independent experiments.

Results presented above suggested that PFO production is regulated by the *C. perfringens agr* locus. A hemoglobin release (Hb) assay [22] was used to examine the kinetics of this regulation. In this assay, the ability of *C. perfringens* supernatants to lyse horse erythrocytes and release Hb is specifically attributable to PFO activity [11], as further supported by our observation that the culture supernatant of a *C. perfringens* strain 13 *pfo*A-null mutant is incapable of inducing Hb release from horse erythrocytes (Fig. 4A).

**Figure 5:**
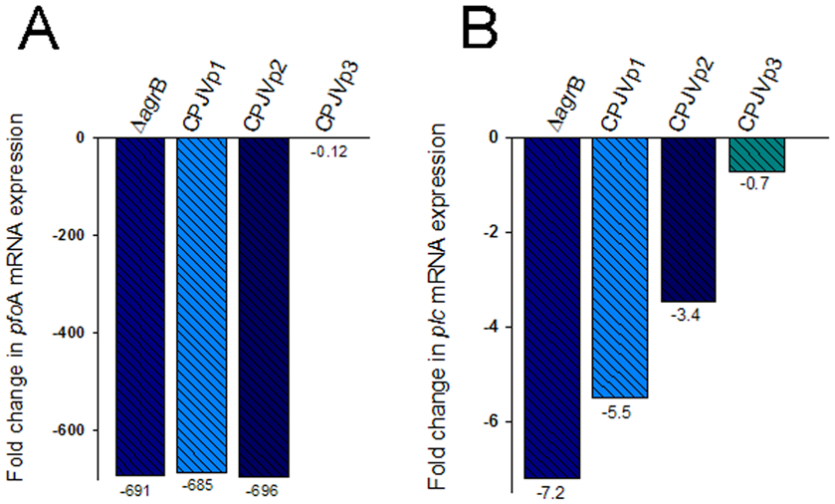
Early transcription of *pfo*A and *plc* genes is regulated by the *C. perfringens agr* locus. Total RNA was extracted from a 2 h TGY culture of the wt strain 13 (S13), CPJV501 (Δ*agr*B), CPJVp1, CPJVp2 or CPJVp3. Quantitative RT-PCR was then performed with 20 ng of each RNA and primers that amplified the (A) *pfo*A gen (pfoAF1 and pfoAR1) or the (B) *plc* gene (cpaF and cpaR). Average *C_T_* values were normalized to the *pol*C gene and the fold differences were calculated using the comparative *C_T_* method (2^−ΔΔ*C*^ *_T_*) [44]. Values below each bar indicate the calculated fold change relative to the wt strain 13. Panels shown are representative of three independent experiments.

Using this horse erythrocyte assay, the appearance of PFO activity in the supernatant of CPJV501 was delayed by 2 h compared to wt strain 13 (Fig. 4A and 4B). This difference was not due to growth differences between CPJV501 and wt strain 13 (Fig. 4C). The absence of PFO activity in the early growth phase supernatants of CPJV501 involved an inhibition of early *pfoA* transcription in CPJV501. Specifically, quantitative RT-PCR analyses revealed that a 2 hour CPJV501 culture contains ∼700-fold less *pfo*A mRNA than does the equivalent culture of wt strain 13 (Fig. 5A).

### Co-culture with Δ*pfo*A strains can restore PFO production by strain 13 *agr*B mutant

**Figure 6:**
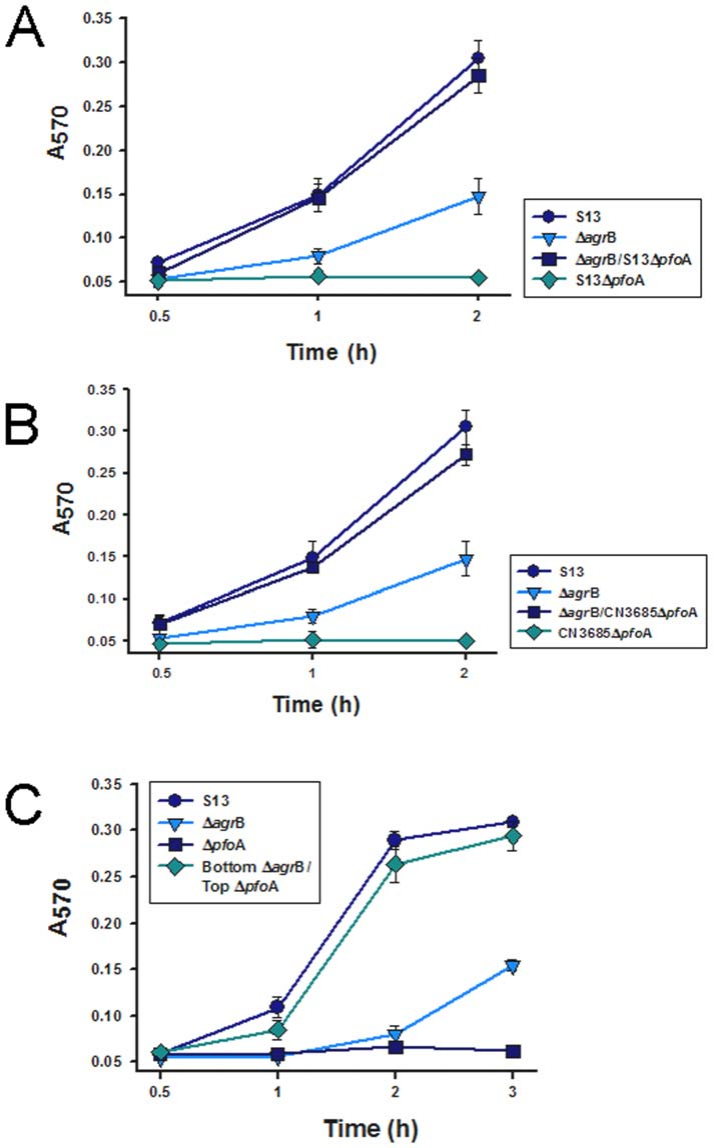
A *C. perfringens* secreted factor(s) regulates PFO production. A and B) Physical complementation of the Δ*agr*B mutant by co-culture with a Δ*pfo*A mutant of strain 13 or CN3685. *C. perfringens* strain 13 (S13), CPJV501 (Δ*agr*B), S13 *pfo*A-null mutant (S13Δ*pfo*A), CPJV501 and S13 *pfo*A-null mutant (Δ*agr*B/S13Δ*pfo*A) or CPJV501 and CN3685 *pfo*A-null mutant (Δ*agr*B/CN3685Δ*pfo*A) were inoculated in TGY and incubated at 37°C for the indicated time. Culture supernatants obtained, at the indicated time point, were incubated (1∶1) with a 1% suspension of horse red blood cells for 30 min at 37°C. Non-inoculated TGY or 0.1% saponin was included as negative or positive control, respectively (not shown). PFO-induced Hb release was detected by obtaining the absorbance at 570 nm (A_570_). C) The physical complementation shown in panels A and B requires a secreted factor to regulate PFO production. Strain 13 (S13), CPJV501 (Δ*agr*B) or S13 *pfo*A-null mutant (Δ*pfo*A) was inoculated in 100 mm tissue culture dishes containing 25 ml of TGY. Another 100 mm tissue culture dish containing a transwell filter device (0.4 µm pore size) received 25 ml of TGY. Then, the S13 *pfo*A-null mutant was inoculated into the top chamber and CPJV501 was inoculated into the bottom chamber of the dish (bottom Δ*agr*B/Top S13Δ*pfo*A) and incubated for the indicated time. Culture supernatants obtained at the indicated time points were incubated (1∶1) with a 1% suspension of horse red blood cells for 30 min at 37°C. PFO-induced Hb release was detected by obtaining the absorbance at 570 nm (A_570_). For all panels, error bars represent the standard error of the mean calculated using data from three independent experiments.

In *S. aureus*, AgrD is a secreted factor that activates the *agr*-mediated regulatory network [23]. In the current study, we found that PFO production by CPJV501 could be restored by coincubating CPJV501 with either a strain 13 *pfo*A-null mutant or a *C. perfringens* type C CN3685 *pfo*A-null mutant, both of which retain an intact *agr* locus (Fig. 6A and 6B). These physical complementation results suggested that toxin regulation by the *agr* locus involves either cell to cell contact or a secreted factor(s). To distinguish between those two possibilities, CPJV501 and strain 13 *pfo*A-null mutant were inoculated into the same 100 mm tissue culture dish but physically separated by a Transwell filter. As shown in Fig. 6C, inoculation of the strain 13 *pfo*A-null mutant into the top well of the dish, and CPJV501 into the bottom well of the dish, restored PFO activity to similar levels as shown by the wt strain 13, i.e. this physical complementation involves a secreted factor produced by both strain 13 and CN3685.

### The *C. perfringens agr* locus also regulates CPA production

**Figure 7:**
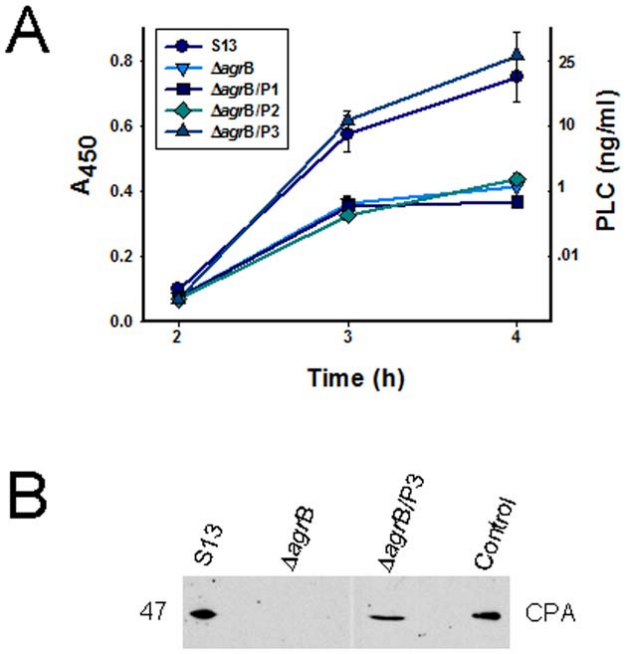
The *C. perfringens agr*B locus regulates CPA production. A) ELISA analyses. Culture supernatants obtained, at the indicated time point, from strain 13 (S13), CPJV501 (Δ*agr*B), CPJVp1 (Δ*agr*B/P1), CPJVp2 (Δ*agr*B/P2), CPJVp3 (Δ*agr*B/P3) or purified CPA was used to coat a 96-well microplate overnight at 4°C. The wells were incubated with a mouse monoclonal anti-CPA antibody followed by a HRP-conjugated anti-mouse antibody. The bound antibody was detected with a TMB substrate solution and the color reaction stopped with sulphuric acid (0.18 M). A_450_ was determined using an ELISA reader. Error bars represent the standard error of the mean calculated using data from three independent experiments. B) Western blot showing the *agr* locus regulates production of CPA. Strain 13 (S13), CPJV501 (Δ*agr*B) or CPJVp3 (Δ*agr*B/P3) was inoculated in TGY and incubated at 37°C for 4 h. Bacteria were then pelleted by centrifugation, resuspended in lysis buffer and sonicated. Equal amount (25 µl) of bacterial lysates was run in a 12% SDS-PAGE, transferred to nitrocellulose membrane and western blotted with a monoclonal anti-CPA antibody. As a control, 25 µl of CPA-containing concentrated supernatant proteins was added to the gel. The expected molecular weight in kDa of CPA is shown at the left. Shown is a representative figure of three independent experiments.

Relative to wt strain 13, production of CPA in culture supernatants was also reduced in 2 h cultures of CPJV501, as detected by an ELISA assay (Fig. 7A). Western blot analyses of bacterial lysates demonstrated that this reduction of CPA supernatant levels was due to decreased intracellular production of CPA by CPJV501, rather than impaired CPA secretion (Fig. 7B). qRT-PCR analyses demonstrated that this reduced CPA production involved transcriptional regulation; compared to wt strain 13, levels of *cpa* mRNA were reduced about 7-fold in 2 hour cultures of this *agr*B mutant (Fig. 5B). Together, these results indicated that the *agr* locus is involved in regulating *pfo*A and *cpa* transcription, and thus PFO and CPA production, during the early logarithmic growth phase.

### Evidence that *C. perfringens agrB* and *agrD* gens are co-transcribed in an operon

In *S. aureus*, the *agr* locus is encoded by an operon consisting of four genes, *agr*B, *agr*D, *agr*C, and *agr*A [23]–[25]. The *agr*A and *agr*C genes encode a response regulator and histidine kinase, respectively, of a two-component regulatory system (TCRS). This TCRS responds to a small peptide named an autoinducer (AI), which is encoded by the *agr*D gene. The *agr*B gene encodes the enzyme cleaving and modifying the AI [24].

To confirm that the *C. perfringens agr* locus contributes to early regulation of PFO and CPA expression in strain 13, and to assess whether *agrB* and *agrD* are expressed by *C. perfringens* as part of an operon, the *agr*B gene alone, the *agr*B and *agr*D genes alone, or the *agr*B, *agr*D and two ORFs (CPE1563 and CPE1562, which are annotated as encoding hypothetical proteins) that lie upstream of *agr*B were cloned into the *E. coli-C. perfringens* shuttle vector pJIR750, creating the plasmids P1, P2 or P3 respectively (Fig. 3C). Those plasmids were then individually electroporated into CPJV501 to create the strains CPJVp1, CPJVp2 or CPJVp3. PCR analyses confirmed the genotype of these CPJV501 complementing strains (Fig. 3B).

Neither CPJVp1 (encoding *agr*B alone) nor CPJVp2 (encoding *agr*B and *agr*D) were able to restore PFO or CPA production (Figs. 4B and 7A). However, complementation with CPJVp3 (encoding CPE1563, CPE1562, *agr*B and *agr*D) did restore 2 h supernatant PFO activity and CPA levels to approximately those found in culture supernatants of the wt strain 13 (Figs. 4B and 7A). qRT-PCR analyses confirmed that 2 h cultures of CPJVp3 exhibited similar levels of *pfo*A and *cpa* mRNA as found in 2 h cultures of the wt strain 13 (Fig. 5). In contrast, CPJVp1 and CPJVp2 exhibited substantially less, if any, complementation in these qRT-PCR analyses.

**Figure 8:**
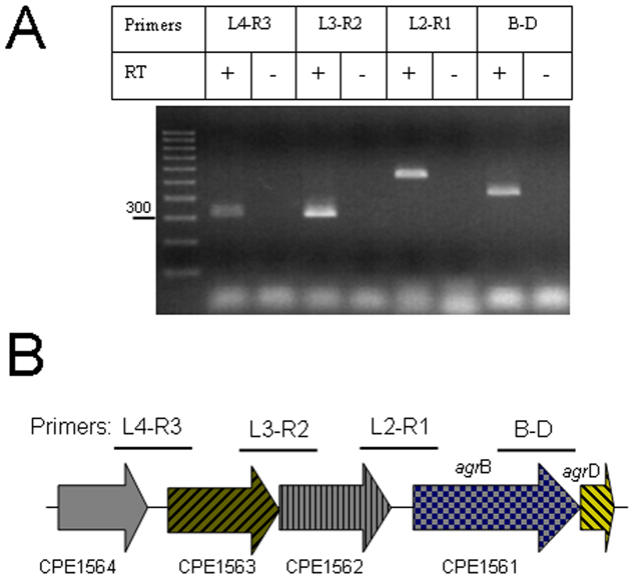
Organization and RT-PCR analysis of the *agr* operon. A) RT-PCR reactions were performed with 50 ng of RNA extracted from an overnight TGY culture of the wt strain 13. RT-PCR reactions included (+) or not (−) retrotranscriptase (RT). The following pair of primers were used to detect mRNA transcripts from every two-adjacent ORF's, agr104L and agr103R (L4-R3, which should generate a 321 bp PCR product), agr103L and agr102R (L3-R2, which should generate a 315 bp PCR product), agr102L and agr101R (L2-R1, which should generate a 420 bp PCR product) or agr101L and agrDR (B–D, which should generate a 520 bp PCR product). A 100-bp DNA ladder is shown at left. B) Schematic representation of the *agr* locus showing primers used for RT-PCR reactions.

Results presented above suggested that *agr*B and *agr*D may be transcribed as an operon that also includes the two upstream ORFs CPE1563 and CPE1562, which are annotated as encoding hypothetical proteins (Fig. 8). To assess further whether *agrB* and *agrD* might be transcribed as part of an operon, RT-PCR analyses were performed using RNA extracted form the wt strain 13. These RT-PCR analyses also used primers that would produce a product only if every two ORF's from CPE1564 through *agr*D are co-transcribed. Fig. 8 results showed evidence for significant levels of co-transcription of CPE1563 and CPE1562, CPE1562 and *agr*B (CPE1561), and *agr*B and *agr*D, strongly suggesting that all four of these genes are co-transcribed in an operon. Interestingly, mRNA transcript was also detected for CPE1564 and CPE1563, but the signal was less intense than for the other transcripts, possibly suggesting a weak promoter can also independently co-transcribe these two ORFs.

## Discussion

This study reports the development of a simple EZ-Tn*5*-based approach for random mutagenesis in *Clostridium perfringens*. All screened EZ-Tn5 mutants obtained by this method contained only a single transposon insertion and were stable over at least 10 sequential overnight culturings. This new approach produced mutants at high efficiency, i.e., 11,200 CFU/µg DNA for strain 13. This mutant yield was 46-fold higher than recently reported for phage Mu-based random mutagenesis of a *C. perfringens* strain 13 derivative [15], which is consistent with previous reports comparing mutant yields for other bacteria when using EZ-Tn5 vs. phage Mu-based random mutagenesis approaches (www.epibio.com).

Besides better efficiency, EZ-Tn*5* random mutagenesis possesses a second advantage over the phage Mu-based system. When applied to *C. perfringens*, phage Mu-based transpositions favored insertion into rRNA genes, with nearly 45% of the phage Mu-based *C. perfringens* mutants carrying an insertion into a rRNA gene [15]. Since another 12% of those phage Mu-based mutants carried an insertion into an intergenic region, only ∼45% of the *C. perfringens* mutants in that phage Mu-based library had the desired outcome, i.e., a transposon insertion into a protein-encoding ORF. While the EZ-Tn*5* transposon exhibited somewhat higher insertion rates into *C. perfringens* rRNA genes than would be expected by mere chance, this preference was much less than observed for phage Mu-based random mutagenesis. Specifically, only 18% of the screened EZ-Tn*5 C. perfringens* mutants carried a transposon insertion into a rRNA gene (rRNA genes represent about 1.5% of total genes in *C. perfringens*). Since none of the EZ-Tn*5 C. perfringens* strain 13 mutants happened to carry a transposon insertion in an intergenic region (although limited intergenic EZ-Tn*5* insertion was observed with another *C. perfringens* strain, data not shown), ∼73% of the transposons in the screened *C. perfringens* strain 13 EZ-Tn*5* carrying mutants had single insertions in a protein encoding gene.

The current study then directly demonstrated the utility of the new EZ-Tn*5* random mutagenesis system by identifying a new locus involved in controlling early log-phase production of α toxin and PFO by *C. perfringens* strain 13. Prior to the current work, regulation of PFO and α toxin expression in strain 13 was known to involve a classical bacterial two component regulatory system named VirS/VirR, where VirS is the membrane sensor and VirR is the transcriptional regulator [11], [17], [18], [26]–[30]. When phosphorylated, the VirR protein binds directly to VirR boxes located upstream of the *pfoA* gene encoding PFO. However, VirR boxes are not present upstream of the *plc* gene encoding α toxin [31]–[34]; instead, a regulatory RNA (named VR-RNA), whose transcription is itself regulated by VirS/VirR, is involved in control of α toxin expression [28]. In addition, previous studies have implicated the LuxS quorum sensing system in the regulation of α toxin and PFO expression by strain 13 [35].

The current study reveals a new level of complexity in the regulation of α toxin and PFO expression by strain 13. Specifically, the current results demonstrated that the early log-phase regulation of α toxin and PFO expression by this *C. perfringens* strain involves a locus containing ORFs with homology to *S. aureus agrB* and *agrD*. A recent bioinformatics search had identified the presence of *agr* ORFs in many firmicutes, including *C. perfringens* [21], but it had not yet been evaluated whether this system is functional or important for regulating *C. perfringens* virulence factor expression. As mentioned in the Results, a similar *agr* locus is well-established in quorum sensing regulation of *S. aureus* virulence, where the *agr* locus controls expression of several toxins, as well as some surface virulence factors [20]. A similar *agr* locus was also recently implicated in *Listeria monocytogenes* virulence, where *agrD*-dependent quorum sensing regulates biofilm formation, Caco-2 cell invasion and mouse virulence [36].

In *S. aureus*, the *agr* locus is a four gene operon transcribed primarily from a promoter named P2 [20]. This *S. aureus agr* operon encodes for a TCRS (that includes the AgrA transcriptional regulator and the AgrC membrane sensor), the agrD signaling peptide, and an AgrB transmembrane protein involved in AgrD processing. Once activated and secreted, extracellular AgrD binds to (and activates) AgrC, which then phosphorylates AgrA. The phosphorylated AgrA then binds to P2 and to another promoter named P3 [25], [37]. This P3 binding leads to production of a regulatory RNA (named RNAIII) encoded by a gene adjacent to the *agr* locus. RNAIII then modulates expression of several exotoxins and surface proteins.

The *agr* systems of *C. perfringens* and *S. aureus* apparently share some similarities. Our results clearly demonstrated that, as in *S. aureus*, the *agr* locus (via a secreted factor) transcriptionally regulates toxin production by *C. perfringens* strain 13. Since the *agr* locus is involved in controlling early log-phase expression of α-toxin and PFO and these two toxins are important for *C. perfringens*-induced gas gangrene [12], [38], [39], the *agr* locus may play an important role in *C. perfringens* virulence, although this needs to be experimentally confirmed. Another similarity, revealed by our RT-PCR and complementation results, is that both *C. perfringens* and *S. aureus* co-transcribe *agrB* and *agrD* as part of an operon.

There also appear to be some differences between the *agr* systems of *S. aureus* versus *C. perfringens.* One variation concerns the arrangement of the *agrB* and *agrD* genes within the *agr* operon. In *S. aureus*, *agrB* and *agrD* are the first two transcribed genes in the operon [20], [25], but in *C. perfringens* these two *agr* genes appear to be downstream of other genes in the operon. It is unclear whether the two ORFs adjacent to *agrB* and *agrD* in *C. perfringens* encode a TCRS, as in *S. aureus*, although those two ORFs co-transcribed with *agrB* and *agrD* in the *C. perfringens agr* operon are currently annotated as encoding “hypothetical proteins” and thus do not possess obvious characteristics of a TCRS. However, future studies should evaluate this possibility. A final difference between the *agr* systems of *C. perfringens* versus *S. aureus* is that the RNAIII-encoding gene lies in close proximity to the *agr* operon in *S. aureus* [25], but no readily identifiable homolog of the RNAIII-encoding gene is located near the *C. perfringens agr* operon or is readily identifiable elsewhere in the strain 13 genome.

If the two upstream ORFs co-transcribed with the *agrB* and *agrD* genes in *C. perfringens* do not encode a TCRS, sorting out the signaling cascade may require some effort. The strain 13 genome contains 28 known or putative histidine kinase sensors [40], which coupled with one or more of the 20 known or putative response regulators, could mediate AgrD signaling. Whether the *C. perfringens agr* locus acts via the VirS/VirR TCRS, which is known to regulate α toxin and PFO production, should be assessed. If *C. perfringens* resembles *S. aureus* by using its Agr system to upregulate the production of a regulatory RNA, that regulatory RNA should be identified. A previous study [28] identified a *C. perfringens* regulatory RNA named VR-RNA that is involved in strain 13 production of α toxin. However, VR-RNA alone would not appear to readily explain *agr*-mediated signaling since, this regulatory RNA is not known to control PFO production [28], yet our *agrB* mutant cannot produce PFO during early log-phase growth. Therefore, further studies might evaluate whether several other putative regulatory RNAs of *C. perfringens* [18] mediate the *agr* locus signal. Also, sorting out the hierarchy of toxin expression control between the *agr* locus, LuxS and the VirS/VirR TCRS will require further studies to fully understand how *C. perfringens* regulates production of its toxins. Additionally, future studies should also examine whether expression of other toxins produced by some *C. perfringens* strains are regulated by the Agr system. Finally, it would be interesting to identify the environmental cues that signal the onset of *agr* operon transcription in *C. perfringens*. While many additional studies are clearly needed to fully understand it roles in *C. perfringens*, linkage of the *agr* locus to PFO and α toxin production opens a new chapter towards understanding toxin gene regulation by the important pathogen *C. perfringens*.

## Materials and Methods

### Strains and bacterial culture media

Strain 13, a genome-sequenced, highly transformable *C. perfringens* type A strain [40] was used for transposon mutagenesis experiments. A strain 13 *pfo*A-null mutant and CN3685 *pfo*A-null mutant were constructed using our previously described Targetron® technology [6], [8]. The bacterial culture media used throughout this study included FTG (fluid thioglycolate medium; Difco Laboratories), TGY (3% tryptic soy broth [Becton-Dickinson]; 2% glucose [Sigma Aldrich], 1% yeast extract [Becton-Dickinson], 0.1% sodium thioglycolate [Sigma Aldrich]), TSC agar medium (SFP agar [Difco Laboratories], supplemented with 0.04% of D-cycloserine [Sigma Aldrich]), Luria-Bertani (LB) broth (1% tryptone [Becton-Dickinson], 0.5% yeast extract, 1% NaCl), LB agar (1.5% agar [Becton-Dickinson]) and brain heart infusion (BHI) agar (Becton-Dickinson). *E. coli* Top10 cells (Invitrogen) were used as the cloning host. When indicated, ampicillin (Amp, 100 µg/ml), erythromycin (Erm [100 µg/ml] or [40 µg/ml]) or chloramphenicol (Cm [15 µg/ml]) was added to the culture medium.

### Construction of the modified EZ-Tn*5* transposon vector and transposome preparation

To modify the EZ-Tn*5*-carrying plasmid pMOD-2 (Epicentre) for use in *C. perfringens*, a single colony of an *E. coli* strain encoding the EZ-Tn*5* pMOD-2 vector was inoculated into 10 ml of LB broth supplemented with ampicillin (LBA) and then incubated overnight at 37°C with shaking (250 RPM). The plasmid was extracted with a QIAprep Spin plasmid extraction kit (Qiagen) and simultaneously digested with EcoRI and HindIII (New England Biolabs). The erythromycin resistance gene (*erm*) from the *E. coli-C. perfringens* shuttle vector pJIR751 [41] was amplified by PCR using JumpStart REDTaq ready mix (Sigma-Aldrich) and primers erm-Fwd-EcoRI and erm-Rev-HindIII (Table 2). The PCR product was run on a 1.5% agarose gel, purified using a QIAquick gel extraction kit (Qiagen), and then simultaneously digested with EcoRI and HindIII. The digested EZ-Tn*5*-carrying pMOD-2 plasmid and the *erm* gene PCR product were ligated overnight at 4°C with T4 DNA ligase (New England Biolab). The resulting plasmid, named pJVTN5, was then transformed into chemically competent *E. coli* Top10 cells (Invitrogen) and inoculated onto LB agar plates supplemented with erythromycin and ampicillin. To prepare the transposome, pJVTN5 was digested with PvuII at 37°C for 1 h (see Fig. 1). The *erm*-carrying EZ-Tn*5* transposon fragment (∼900 bp) was purified from an agarose gel as described earlier and DNA concentration was quantified. Two µl of the *erm*-modified EZ-Tn*5* Transposon DNA (100 µg/ml in TE Buffer [10 mM Tris-HCl (pH 7.5), 1 mM EDTA]) were mixed with 4 µl of the EZ-Tn*5* transposase (Epicentre) and 2 µl of glycerol. The mixture was incubated for 30 min at room temperature, to allow the transposase to stably bind to the *erm*-modified EZ-Tn*5* Transposon DNA; that mixture was then stored at −20°C.

### Transposome electroporation into *C. perfringens* strains

Following a standard procedure [6], [42], 1 µl of the transposome was electroporated into a 4 hour TGY culture of highly transformable *C. perfringens* strain 13 [40]. For this purpose, electrocompetent cells (400 µl) were mixed, in a 0.2 cm electroporation cuvette (Biorad), with 1 µl of the transposome and incubated 5 min at 4°C. Electroporation was performed using a BioRad Gene Pulser™ with pulse controller set at 200Ω, 25 µF and 1.5 kV. Electroporated transposome-containing bacteria were grown in 3 ml of pre-warmed TGY for 3 h at 37°C to allow them complete recovery, plated onto BHI agar plates with erythromycin (40 µg/ml), and incubated at 37°C for 18 h under anaerobic conditions. All colonies were propagated in BHI agar plates with erythromycin (40 µg/ml). To confirm the presence of the *erm* gene, a PCR was performed with 2 µl of cell lysate as DNA template. This PCR used primers erm-Fwd-EcoRI and erm-Rev-HindIII and the following PCR conditions: 1 cycle of 95°C for 5 min, 35 cycles of 95°C for 30 s, 55°C for 45 s, and 68°C for 1 min; and a single extension of 68°C for 10 min.

### Sequencing of the EZ-Tn*5* target gene in selected mutants

After electroporation of the transposon, *C. perfringens* strain 13 Erm-resistant transformants, which were also *erm*-positive by PCR, were randomly chosen for sequencing. Total DNA was extracted and 1 µg of each DNA was mixed with 10 pmol of primers pMOD-SeqFwd or pMOD-SeqRev (Epicentre) and sent for sequencing at the University of Pittsburgh Genomics and Proteomics Core Laboratory. *C. perfringens* sequences flanking the *erm*-carrying transposon were determined using the nucleotide BLAST program on the National Center for Biotechnology Information (NCBI) web site and the J. Craig Venter Institute's Pathema website programs.

### Complementation of the *agrB* mutant

DNA was isolated from strain 13 using a Master Pure™ Gram Positive DNA purification Kit (Epicentre). The primers agrBF and agrBR (Table 2) were added (at a 5 µM final concentration) to a PCR mixture containing 1 µl of purified DNA template and 25 µl 2×Taq mixture (NEB). Those reaction mixtures, with a total volume of 50 µl, were placed in a thermal cycler (Techne) and subjected to the following amplification conditions: 1 cycle of 95°C for 2 min, 35 cycles of 95°C for 30 s, 55°C for 40 s, and 68°C for 3 min, and a single extension of 68°C for 5 min. The PCR products were cloned into a TOPO vector (Invitrogen) and sequenced at the University of Pittsburgh Core Sequencing Facility. Using EcoRI and BamHI, the insert was removed from the TOPO vector and ligated into pJIR750, forming a plasmid named P1 (which is 1072 bp and contains *agrB* and a 403 bp upstream sequence). Using the same method, two other complementing plasmids were created, including P2 (created using agrBF-agrR1 primers and which has a 1230 bp insert containing *agrB* and *agrD*, along with a 403 bp upstream sequence) and P3 (created using agrF1-agrR1 and which has a 2893 bp insert containing *agrB*, *agrD* and two upstream ORFs encoding hypothetical proteins). Plasmids P1, P2 and P3 were separately introduced, by our standard electroporation techniques, into the *agrB* mutant of strain 13. Chloramphenicol (15 µg/ml) resistant transformants were then selected. The resultant transformants were designated CPJVp1, CPJVp2 and CPJVp3.

### Southern blot analyses

*C. perfringens* DNA was isolated using the MasterPure gram-positive DNA purification kit (Epicentre, Wisconsin). Each isolated DNA sample (2.5 µg) was then digested overnight with EcoRI or XbaI, according to the manufacturer's (New England Biolabs) instructions. The digested DNA samples were electrophoresed on a conventional 1% agarose gel, and the separated DNA digestion products were then transferred onto nylon membranes (Roche) for hybridization with an *erm*-specific probe. After hybridization of the *erm* probe, the Southern blots were developed using reagents from the DIG DNA labeling and detection kit (Roche), according to the manufacturer's instruction.

### Hemoglobin (Hb) release assay for PFO activity

*C. perfringens* strain 13 (S13), S13 *pfo*A-null mutant, CPJV501, CPJVp1, CPJVp2, CPJVp3, or a combination of CPJV501 and S13 *pfo*A-null mutant (at a 1∶1 ratio), or CPJV501 and CN3685 *pfo*A-null mutant (at a 1∶1 ratio), were inoculated into 10 ml of FTG and grown overnight at 37°C. An aliquot (100 µl) of this overnight culture was then inoculated into a test tube containing 10 ml of sterile TGY (OD_600_∼0.05) and grown for the indicated times. At each time point, the OD_600_ of the culture was recorded. In other experiments, 100 mm tissue culture dishes (Costar) containing 25 ml of TGY were inoculated (OD_600_∼0.05) with strain 13 or CPJV501 and incubated at 37°C for the indicated times. Another experiment used a 100 mm tissue culture dish containing a Transwell® filter (Costar) and 25 ml of TGY; strain 13 *pfo*A-null mutant was inoculated into the top well of the dish and CPJV501 was inoculated into the bottom chamber of the culture dish (at a 2∶1 ratio). This culture was also incubated at 37°C for different times. The culture supernatant was obtained and filter sterilized using a 0.45 µm filter (Millipore). The Hb release assay was then performed essentially as previously described [43].

### ELISA

*C. perfringens* alpha toxin (1 µg/ml) purchased from Sigma Aldrich, or culture supernatants, were analyzed by ELISA using mouse monoclonal anti-CPA antibody (kindly provided by Dr. Paul Hauer), as previously described [43].

### Western blot

Strain 13 (S13), CPJV501 (Δ*agr*B) or CPJVp3 (Δ*agr*B/P3) was inoculated in 10 ml of TGY and incubated at 37°C for 4 h. Bacteria were then pelleted by centrifugation at 8000×*g* at 4°C for 30 min. The bacterial pellet was resuspended in 500 µl of ice-cold lysis buffer [40 mM Tris-HCl pH 7.5, 100 mM NaCl, 1× protease inhibitors (Roche), 1 mM DTT and 1% Triton X-100] and then sonicated. Equal amounts (25 µl) of bacterial lysates were run in a 12% SDS-PAGE and transferred to nitrocellulose membranes. As control, 25 µl of 100× ammonium sulfate supernatant-concentrated proteins of an overnight culture of strain 13 was also run in the gel. Those membranes were blocked with PBS-Tween 20 (0.05% v/v) and non fat dry milk (5% wt/v) for 1 h and then probed with a mouse monoclonal anti-CPA antibody. Bound antibody was then detected after incubation with a horseradish peroxidase-conjugated secondary anti-species specific antibody and addition of Chemiluminescent Substrate (Pierce).

### RT-PCR and qRT-PCR

Total *C. perfringens* RNA was extracted from 2 ml of an overnight TGY culture by the following procedure. After centrifugation of that culture (10,000×*g* at 4°C), the pellet was resuspended in 200 µl of acetate solution (20 mM sodium acetate [pH 5], 1 mM EDTA, 0.5% sodium dodecyl sulfate [SDS, Bio-Rad]). The suspension received 200 µl of saturated phenol (Fisher scientific) and was thoroughly resuspended before incubation at 60°C in a water bath with vigorous shaking for 5 min. After centrifugation (10,000×*g* at 4°C for 5 min), the nucleic acid-containing supernatant received cold ethanol and that sample was mixed well. The mixed sample was centrifuged (10,000×*g* at 4°C) for 5 min to obtain the RNA pellet. This pellet was washed two times with cold 70% ethanol and finally resuspended in 100 µl of DNase-free, RNase-free water. All RNA samples were additionally treated with 2 U of DNase I (Promega) at 37°C for 30 min. To stop DNase I activity, DNase I inhibitor (Promega) was added to the reaction tube. RNA was quantified by absorbance at 260 nm and stored in 50 ml aliquots at −80°C.

RT-PCR reactions were then performed on those DNase-treated RNA samples using the AccesQuick RT-PCR system (Promega). Briefly, 50 ng of each RNA sample were reversed-transcribed to cDNA at 45°C for 45 min and then used as template for PCR reactions (denaturing at 94°C for 1 min, annealing at 55°C for 1 min and extension at 72°C for 1 min) with the gene-specific primers. Control RT-PCR reactions were similarly performed, except for the omission of reverse transcriptase.

Quantitative RT-PCR (qRT-PCR) was performed using the iScript One-Step RT-PCR kit with SYBR Green (Bio-Rad) and the iCycler thermal cycler with a 96-well reaction module (Bio-Rad). qRT-PCR reactions were performed in triplicate with 20 ng of total RNA, 500 nM concentration of each primer (Table 2) and the following conditions; 1 cycle at 50°C for 30 min, 1 cycle at 95°C for 10 min and 40 cycles of 95°C for 15, 55°C for 1 min. Melting curves were generated by a cycle of 95°C for 1 min, 55°C for 1 min and 80 cycles of 55°C with 0.5°C increments. The relative quantitation of mRNA expression was normalized to the constitutive expression of the housekeeping *pol*C gene and calculated by the comparative *C_T_* (2^−ΔΔCT^) method [44].

### Note added during revision

During preparation of the revised version of this paper, Ohtani et al. (2009) published work also implicating the *C. perfringens agr* locus in control of PFO and CPA production by strain 13 (Ohtani, K., *et al.* J. Bacteriology. 2009. 191(12):3919–27).
